# ADNP Regulates Cognition: A Multitasking Protein

**DOI:** 10.3389/fnins.2018.00873

**Published:** 2018-11-26

**Authors:** Illana Gozes

**Affiliations:** Laboratory for Molecular Neuroendocrinology, Department of Human Molecular Genetics and Biochemistry, Sackler Faculty of Medicine, Sagol School of Neuroscience and Adams Super Center for Brain Studies, Tel Aviv University, Tel Aviv, Israel

**Keywords:** ADNP (activity dependent neuroprotective protein), ADNP gene, cognition, protein interaction, neurodegenaration

## Introduction

With the advantage of rapid progress of DNA/RNA sequencing techniques, it has become feasible to identify the cause of developmental disorders encompassing intellectual disabilities to single *de novo* mutated genes (e.g., Larsen et al., 2016; Deciphering Developmental Disorders, 2017; Stessman et al., 2017). It is my opinion that we should study in depth, the leading identified genes, to acquire better understanding of the molecular basis for human cognitive functions. Furthermore, from a translational science point of view, understanding genes regulating cognition will facilitate drug development to currently untreatable devastating disease, which hamper cognition. Here, I focus on activity-dependent neuroprotective protein (ADNP) (Gozes et al., 2018) showing a tight association with cognition, and in my opinion, a key gene regulating cognitive functions.

## Activity-dependent neuroprotective protein (ADNP)

Our original studies identified vasoactive intestinal peptide (VIP) (Bodner et al., 1985) as a gene/protein highly expressed at the time of synapse formation (Gozes et al., 1987), which was translated to VIP-associated neuroprotection (Brenneman and Eiden, 1986) and VIP-related synaptogenesis, through astrocyte activation (Blondel et al., 2000). Astrocyte activation entailed secretion of protein growth factors, leading to the cloning/discovery of ADNP and its active neuroprotective site, NAP (NAPVSIPQ) (Bassan et al., 1999; Zamostiano et al., 2001). To elucidate ADNP's *in vivo* activity we knocked out the *ADNP* gene and showed that this gene is essential for neural tube closure and brain formation (Pinhasov et al., 2003). At the single cell level, ADNP is found in the nucleus and upon neuronal maturation, the protein is found also in the cytoplasm with specific RNA silencing resulting in loss of microtubules/loss of neurites (Mandel et al., 2008). While complete knockout of ADNP is lethal, haploinsufficient (heterozygous) mice survive, showing cognitive impairment (Vulih-Shultzman et al., 2007). Further results indicate microtubule insufficiency, reduced axonal transport (Amram et al., 2016) and reduced dendritic spines (Hacohen-Kleiman et al., 2018) in the *Adnp*^+/−^ mice. These findings are in line with patient results showing intellectual disabilities in case of ADNP gene heterozygous microdeletion or truncating mutation (Borozdin et al., 2007; Vandeweyer et al., 2014; Huynh et al., 2018). Given the fact that ADNP is a large protein it includes many identified signature motifs for macromolecular interactions and here I will concentrate on the ADNP motifs, protein interactors and the strong link to cognition.

## ADNP binding motifs

ADNP contains a nuclear localization signal (NLS) and a homeobox domain profile (Bassan et al., 1999; Zamostiano et al., 2001). ADNP has heterochromatin protein 1 (HP1) binding domains (Mandel et al., 2007; Mosch et al., 2011) and interacts with DNA in a sequence-specific manner, as well as with HP1 (Mandel et al., 2007; Mosch et al., 2011) and chromodomain-helicase-DNA-binding protein 4 (CHD4) (Ostapcuk et al., 2018). ADNP was discovered to bind and affect the SWItch/Sucrose Non-Fermentable (SWI/SNF) chromatin remodeling complex (Mandel and Gozes, 2007) also associated with alternative RNA splicing (Schirer et al., 2014). The DNA/chromatin binding characteristics have been further implicated in promoter/control gene specific regions for ADNP binding and direct regulation of RNA expression (Mandel et al., 2007; Dresner et al., 2012). Complete gene array analysis, RNA-seq and high-throughput platform BioMark™ HD System (Fluidigm) identified hundreds of ADNP regulated transcripts (Mandel et al., 2007; Amram et al., 2016; Hacohen-Kleiman et al., 2018) suggesting a master gene regulator function.

In the cytoplasm, ADNP was found to bind eukaryotic initiation factor 4E (Eif4e), implicating an involvement in the protein translation machinery (Malishkevich et al., 2015) and the autophagy complex, by direct binding to microtubule associated protein 1 light chain 3B (LC3B) (Merenlender-Wagner et al., 2015; Sragovich et al., 2017). ADNP provides potent neurotrophic/neuroprotective activity that can be attributed, at least in part, to NAP (davunetide, AL-108 or CP201 (Bassan et al., 1999; Gozes et al., 2018). In short, the SIP domain in NAP interacts with microtubule end binding proteins (EB1 and EB3) enhancing ADNP (Esteves et al., 2014) and tau (Ivashko-Pachima et al., 2017) interaction with microtubules. This SxIP (SKIP) domain in NAP further protects against deficits in axonal transport occurring because of ADNP deficiency (Amram et al., 2016) and NAP enhances ADNP interaction with the autophagosome membrane protein LC3B (Merenlender-Wagner et al., 2015). *In vivo* NAP restores multiple anomalies caused by ADNP haploinsufficiency (Vulih-Shultzman et al., 2007; Hacohen-Kleiman et al., 2018). Lastly, our original studies have shown that ADNP has a glutaredoxin active site (Bassan et al., 1999).

## Proteins interacting with ADNP

**Figure 1:**
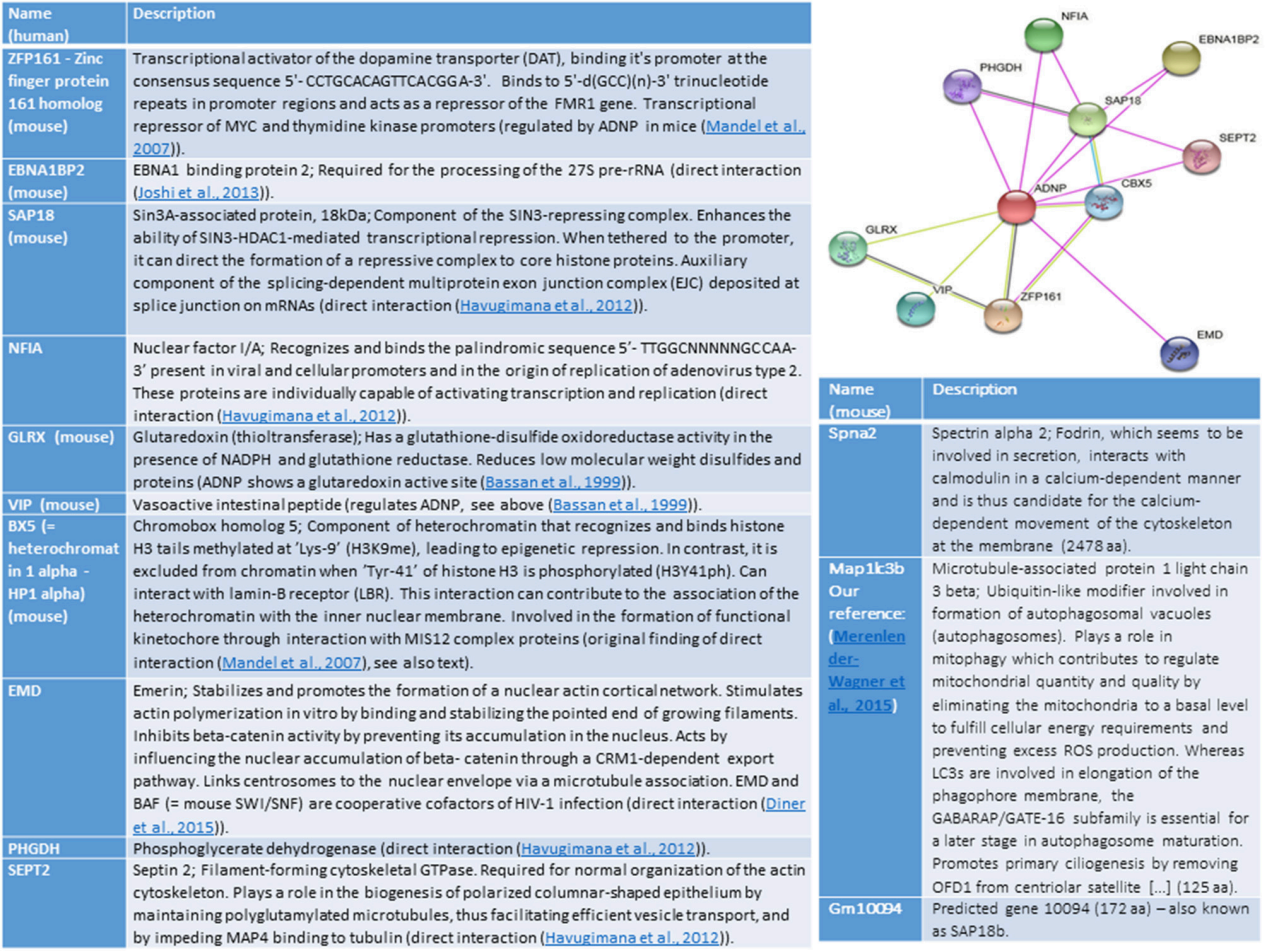
The illustration shows ADNP interactions (String analysis, https://string-db.org/cgi/network.pl?taskId=P3YfAA8ZrZzy). Edges represent protein-protein associations, Known-interactions from curated databases are shown in light blue, experimentally determined are in magenta. Predicted Interactions include, gene neighborhood (green) gene fusions (red) gene co-occurrence (violet), textmining (light green), co-expression (black) and protein homology (light violet). References cited in the figure and not cited above include (Havugimana et al., 2012; Joshi et al., 2013; Diner et al., 2015).

Ten ADNP-interacting proteins were identified when analyzing (string) for human genes and 9 proteins when searching for mouse associations, with 6 overlapping proteins (Figure 1). Some of these proteins are described in the section above. The common mouse and human proteins, not described above, include ZFP161–Zinc finger protein 161 homolog (mouse), which is a transcriptional activator of the dopamine transporter (DAT). ZFP161 also acts as a repressor of the FMR1 gene (fragile X syndrome). We have originally shown that ZF5 is linked to regulation by ADNP in the developing mouse embryos (Mandel et al., 2007). Another shared mouse and human protein, EBNA1BP2 is linked to early onset Alzheimer's disease (https://www.malacards.org/card/early_onset_familial_alzheimer_disease). A third one, SAP18 enhances the ability of SIN3-HDAC1-mediated transcriptional repression. When tethered to the promoter, it can direct the formation of a repressive complex to core histone proteins. SAP18 is an auxiliary component of the splicing-dependent multiprotein exon junction complex (EJC) deposited at splice junction on mRNAs, and our laboratory has shown interaction of ADNP with the RNA splicing machinery (Schirer et al., 2014). ADNP-interacting proteins described for either human or mouse, include actin-interacting proteins (EMD – nuclear), SEPT2—cytoplasmic and Spna2—associated with the cytoskeleton. Other interacting proteins are NFIA, linked to viral infection, PHGDH, linked to cytoplasmic energy metabolism and SAP18b (Gm10094, http://www.informatics.jax.org/marker/MGI:1277978).

## ADNP and cognition

Our studies showed that VIP and VIP derivatives protected against Alzheimer-like pathology (Gozes et al., 1996, 1999). Furthermore, the VIP receptor, VPAC2, controlling ADNP expression (Zusev and Gozes, 2004), has been linked to schizophrenia and autism spectrum disorders (Vacic et al., 2011; Ago et al., 2018) and VIP regulates ADNP expression *in vivo* (Giladi et al., 2007). Our discovery of the requirement of ADNP for brain formation (Pinhasov et al., 2003) coupled with the finding that a major phenotypic outcome of ADNP haploinsufficency in mice leads to cognitive impairments, placed ADNP as a key regulatory gene for brain function (Vulih-Shultzman et al., 2007). The direct involvement of ADNP in cognitive function was reported in our 2007 *Adnp* haploinsufficient mouse model (Vulih-Shultzman et al., 2007) coupled with a paper showing that deletion in the chromosomal area including ADNP [20q12–13.2 (Zamostiano et al., 2001)] specifically, 20q13.13–q13.2 (Borozdin et al., 2007) resulted in developmental delays and intellectual disabilities in humans. Both animal studies (Malishkevich et al., 2015; Amram et al., 2016; Hacohen-Kleiman et al., 2018) as well as the human studies were repeated and extended showing axonal/synaptic/behavioral dysfunctions at the mouse level (Amram et al., 2016; Hacohen-Kleiman et al., 2018) mirroring the human situation when the *ADNP* gene is partially deleted (Huynh et al., 2018) or pathologically mutated (Helsmoortel et al., 2014; Vandeweyer et al., 2014; Gozes et al., 2015, 2017a,b, 2018; Arnett et al., 2018; Van Dijck et al., 2018). Over the last 4 years it became apparent that the mutated *ADNP* gene is consistently reported as one of the most frequent causes of syndromic autism and intellectual disability (Helsmoortel et al., 2014; Larsen et al., 2016; Deciphering Developmental Disorders, 2017; Stessman et al., 2017).

Notably, the involvement of ADNP in cognitive performance is not limited to the *ADNP* syndrome but is extended to schizophrenia (Merenlender-Wagner et al., 2015) and Alzheimer's disease (Malishkevich et al., 2016) with *ADNP* transcripts dysregulated in lymphocytes in both diseases and with ADNP blood levels correlating with intelligence (Malishkevich et al., 2016). Thus, the current opinion combines mechanisms to cognitive protection.

Furthermore, NAP activity is not limited to the mouse model, but has shown efficacy in amnestic mild cognitive impairment patients, prodromal to Alzheimer's disease (protecting short term memory) and in schizophrenia patients (protecting functional activities of daily living as reviewed; Magen and Gozes, 2013, 2014). Currently, Coronis Neurosciences (www.coronisns.com) is developing NAP (CP201) for the ADNP syndrome.

## Conclusions

This opinion article connects ADNP to a network of proteins linked with cognitive abilities. As many cases within the autism spectrum disorders and developmental disorders are caused by single gene mutations, it is of great interest to understand the protein interactions to get a comprehensive understanding of the molecular basis of cognition. Specifically, in the case of ADNP, which has been correlated with intelligence in the developing child and in the elderly, in autism spectrum disorders, the ADNP syndrome, in Alzheimer's disease and cognitive impairments associated with schizophrenia. The case of ADNP is unique with the identification of its active neuroprotective site, NAP. Outlined above are protein interacting with the multitasking ADNP, which are linked in part to neurodevelopment and cognition. For example, mutations in CHD4 (OMIM # 617159) cause neurodevelopmental delays, chromatin remodelers have been associated with cognition (Wenderski and Maze, 2016), Eif4e has been tightly linked with autism (St Clair and Johnstone, 2018) and autophagy with autism, brain degeneration and schizophrenia (Sragovich et al., 2017). Finally, ADNP's interaction with cytoskeletal proteins shapes the synapse and contributes to brain plasticity (Gozes et al., 2018; Hacohen-Kleiman et al., 2018). Understanding ADNP multitasks and interacting proteins, will allow the development of NAP and pipeline for other related diseases, syndromes affected by single gene mutations and allow cross-over drug repositioning clinical developments for the benefit of the cognitively impaired patient, families and society at large.

## Author contributions

The author confirms being the sole contributor of this work and has approved it for publication.

### Conflict of interest statement

IG is the Chief Scientific Officer of Coronis Neurosciences, developing CP201 (under patent protection and license from Ramot at Tel Aviv University).
